# Twelfth Annual Meeting of American Medical Association

**Published:** 1859-06

**Authors:** 


### TWELFTH ANNUAL MEETING OF THE AMERICAN MEDICAL ASSOCIATION.

### ABSTRACT OF PROCEEDINGS.

LOUISVILLE, May 3, 1859.

The Association convened in Mozart Hall, at 11 o'clock A. M., and was called to order by the President, Dr. Harvey Lindsly in the chair, supported by Vice Presidents Drs. W. L. Sutton and T. O. Edwards. The other officers present were the Secretaries, Drs. A. J. Semmes and S. M. Bemiss, and Treasurer Caspar Wister.

The President introduced Rev. Stewart Robinson, of Louisville, who opened the proceedings with prayer.

The chair then declared the Association duly organized, and announced as first in order of business, the report of the Committee of Arrangement.

Dr. R. J. Breckenridge, chairman of this committee, reported as follows:

*Mr. President, and Gentlemen of the Association:*

It is my grateful office to greet you on this your twelfth anniversary, and tender you a hearty welcome to the city of Louisville. I do this, sir, in behalf of the physicians and citizens generally—citizens, second to none in their intelligent appreciation of the honor and dignity of the profession, and the worthiness and usefulness of its members;—physicians, second to none in their devotion to the great work in which they are engaged.

We have watched, sir, with interest, the formation and progress of this association. We have noted, with equal gratification, the catholicity of its spirit, and the greatness of its designs. We have seen it, in its brief existence, gather into its fold thousands of members—members from every State of the Republic, and without possessing real legislative powers, exercise a most potent influence for good.

Formed for the advancement of science and art—for the gathering, interchange and diffusion of knowledge—for the promotion of fellowship and harmony in the profession, by drawing closer and closer its members, it has *not* wholly failed in the accomplishment of its aims; and we trust for it a future yet more fruitful—harvests yet more abundant.

Feeling that "it is good for us to be here"—approving thoroughly, cordially, the objects of the association—and believing in its capacity for usefulness—we bid you God speed in your labors, while we heartily welcome you, as honored guests, to our homes.

The Secretary then called the roll.

The President now announced a recess of fifteen minutes, to enable the various State delegations to choose their members for the committee on nominations.

Upon call to order, the following members were reported as the Nominating Committee:

New Hampshire, Dixi Crosby; Massachusetts, Solomon D. Townsend; Rhode Island, J. H. Eldridge; New York, D. M. Reese; New Jersey, A. N. Dougherty; Pennsylvania, R. K. Smith; Delaware, H. F. Askew; Maryland, G. W. Lawrence; District Columbia, Cornelius Boyle; Virginia, L. S. Joynes; North Carolina, Edward Warren, Jun.; South Carolina, J. M. Gaston; Georgia, John W. Jones; Alabama, J. B. Coons; Louisiana, S. O. Scruggs; Tennessee, E. B. Haskins; Kentucky, D. D. Thomson; Ohio, George Fries; Indiana, J. H. Brower; Michigan, William Brodie; Illinois, C. Goodbrake; Missouri, M. L. Linton; Iowa, D. L. McGugin; Wisconsin, C. B. Chapman; Army, Charles S. Tripler.

The President then appointed the following committee on voluntary essays: Drs. L. P. Yandell, of Kentucky, James Bryan, of Philadelphia, and C. G. Comegys, of Ohio.

Dr. R. J. Breckinridge, from the Committee of Arrangements, announced the hours of business from 9 A. M. to 1 P. M., and from 3 P. M. until such hour as the Convention should adjourn upon resolution.

Dr. Harvey Lindsly, the President of the Association, then read his retiring address, which was listened to with marked attention, and was an eloquent tribute to the dignity of the medical profession and the importance of its improvements.

Dr. L. A. Smith, of New Jersey, moved that the thanks of the Association be tendered to the President for his able and eloquent address, and it was ordered to be placed in the hands of the appropriate committee for publication, among the proceedings of the meeting.

Dr. Caspar Wister, chairman of the Committee on Publication, read the annual report, and on motion of Dr. Sayre, of New York, the following resolutions appended to it were adopted:

*Resolved*, That hereafter every paper intended for publication in the Transactions must not only be placed in the hands of the Committee of Publication by the first of June, but it must also be so prepared as to require no material alteration or addition at the hands of the author.

*Resolved*, That authors of papers be required to return their proofs within two weeks after their reception, otherwise they will be passed over and omitted from the volume.

Adjourned until 3 o'clock P. M.

AFTERNOON SESSION, 3 O'CLOCK.

Dr. W. L. Sutton, one of the Vice Presidents, took the chair in the absence of the President.

Dr. D. Meredith Reese, of New York, chairman of the committee on nominations, reported the following officers for the ensuing year:

President—Henry Miller, of Kentucky.

Vice Presidents—H. F. Askew, Delaware; Chas. F. Tripler, U. S. Army; L. A. Smith, New Jersey; Calvin West, Indiana.

Treasurer—Caspar Wister, Pennsylvania.

Secretary—S. M. Bemiss, Kentucky.

Dr. Sayre moved the adoption of the report, which was unanimously agreed to.

Dr. Brainard, of Illinois, moved the appointment of a committee to conduct the newly appointed officers to their respective chairs. The acting President selected Drs. Brainard, of Illinois, Mattingly, of Kentucky, Sutton, of Indiana, McDowell, of Missouri, and R. J. Breckinridge of Kentucky, and they accordingly performed the duties assigned to them.

The newly elected President, on taking the chair, addressed the convention in substance as follows:

*Gentlemen of the American Medical Association:*

I am wholly at a loss to command language to express the deep sense of obligation put upon me by calling me to the presidency of your association. It is an honor any man may well be proud of, and although I admit, in all sincerity, that you might without difficulty have selected an individual more worthy the position, I may be allowed to say you could not have conferred it upon one who would prize it more highly, or cherish it longer with the most grateful recollection. I do esteem it the greatest honor ever conferred upon me by the profession that I love, and to which I have devoted a long life; nay, more, it is the greatest honor that could be conferred upon any man, by the medical or any other profession, in this or any other country; for any decoration of honor, or any mark of approbation conferred by a crowned head, I should regard as a bauble in comparison. Who are you, gentlemen, when rightly considered? You are the rightful representatives of the great American Medical Profession—an army forty thousand strong, and a body of men, no matter what captious criticism may say in disparaging comparison with the European branch of the profession, in my humble judgment, far superior to the same number of medical men to be found in any quarter of the globe. Although, as a body, you may not be so learned, so critically and nicely framed in all the minutiæ of the profession, yet for strength, integrity and precision in all the great principles guiding to a successful combat with disease, this body is equal if not superior to that of any kingdom of continental Europe.

To be called to the presidency of such a body of men, is, in my sober judgment, the greatest compliment that could be conferred on mortal man, provided that man is a devotee of medicine, who has given his whole mind, soul, heart and strength, individually, to the profession, and has that high regard for it which will not suffer any less noble pursuit to interfere with the daily though laborious duties of the profession.

Coming so recently from a sick bed, and still enfeebled in health, I beg to be excused from further remarks, and desire you to accept this brief and imperfect acknowledgement of the distinguished honor conferred upon me, instead of what, under other circumstances, I might be disposed to say.

Dr. J. B. Lindsly, of Tennessee, offered the following:

*Resolved*, That a committee of three be appointed by the chair, to inquire into and report upon the propriety of dividing the association into sections for the purpose of performing such parts of its scientific labors as may relate to particular branches of medicine and surgery.

Dr. Brodie moved its reference to the nominating committee.

Dr. Brainard explained at some length the object of the resolution of inquiry, and urged its adoption as the means of giving more effect and usefulness to the proceedings of the association, the reports of which had heretofore gone out unmatured, in consequence of the want of concentrated action.

A motion by Dr. Sayre to lay the motion on the table was negatived, and the motion of Dr. Lindsly was then adopted.

The chair appointed as the committee, Dr. Lindsly of Tennessee, Dr. Brainard of Illinois, Dr. G. C. Blackman of Ohio.

Dr. Davis moved that no person should be permitted to speak more than twice on the same subject, or more than ten minutes at one time, except by consent of the association, which was adopted.

The committee on prize essays was called, but the chairman being temporarily absent, was postponed.

The committee on medical education failed to report.

The committee on medical literature failed to report.

A letter from Dr. J. G. F. Holston, of Ohio, chairman of the special committee on the microscope, was read, reported progress and begging a continuance for more extended investigation, which was referred to the committee on nominations.

A letter from Dr. Stephen Smith of New York, from the special committee on medical jurisprudence, had the same reference.

The special committee on quarantine was not ready to report.

Dr. Mattingly of Kentucky, from the special committee on diseases and mortality of boarding schools, asked a continuance until next year, in order to obtain further information requisite to the full investigation of the important subject. The request was referred to the committee on nominations.

The special committee on surgical operations for the relief of defective vision, on milk sickness, and on the blood corpuscle, had the same reference.

The report from the committee on medical ethics was read, and such portion of it as related to the action of the Dubuque Medical Society in the case of an expelled member, was, on motion of Dr. T. O. Edwards, made the special order for 12 M. to-morrow.

The special committee on government meteorological reports made a report, written by Dr. R. H. Coolidge, of the U. S. Army, but read by Dr. Paul F. Eve, of Tennessee, which was referred to the committee on publication.

The committee appointed in May, 1857, on criminal abortion, submitted a report written by Dr. Storer, of Boston, which was read by Dr. Blatchford, of New York, and referred to the committee on publication. The following resolutions appended to this report were unanimously adopted:

*Resolved*, That while physicians have long been united in condemning the act of producing abortion, at every period of gestation, except as necessary for preserving the life of either mother or child, it has become the duty of this association, in view of the prevalence and increasing frequency of the crime, publicly to enter an earnest and solemn protest against such unwarrantable destruction of human life.

*Resolved*, That in pursuance of the grand and noble calling we profess—the saving of human lives—and of the sacred responsibilities thereby devolving upon us, the association present this subject to the attention of the several legislative assemblies of the Union, with the prayer that the laws by which the crime of procuring abortion is attempted to be controlled, may be revised, and that such other action may be taken in the premises as they in their wisdom may deem necessary.

*Resolved*, That the association request the zealous co-operation of the various State Medical Societies in pressing the subject upon the legislatures of their respective States, and that the president and secretaries of the association are hereby authorized to carry out by memorial these resolutions.

The convention then adjourned till to-morrow morning, at 9 o'clock.

SECOND DAY.

WEDNESDAY, May 4, 1859.

The president, Dr. Miller, called the association to order at 9 o'clock.

Dr. D. Meredith Reese, chairman of the committee on nomination, called attention to the fact that the committee could not act definitely until the place for next year's meeting should be designated. He stated also that the Medical State Society of Connecticut had requested that an amendment to the constitution, proposed two years since, should be taken from the table, relative to the time of meeting.

It was moved by Dr. Blatchford, and seconded by Dr. Sayre, that the amendment to the third article of the constitution be taken up, which proposes to add after the words "first Tuesday of May," the words "or first Tuesday of June," and after the words "shall be determined," add the words "with the time of meeting."

The amendment was adopted by a constitutional vote.

Dr. D. M. Reese also stated that the Connecticut State Society had extended a pressing invitation to the association to hold its next meeting at New Haven, which invitation was referred to the committee on nominations.

Dr. Reese also called attention to the necessity of some radical change in the mode of appointing committees to prepare treatises on scientific subjects to be reported at the annual meetings. It had been seen, that on yesterday, a large majority of the committees made no reports, and did not even see proper to send in any communication explanatory of delay. The difficulty heretofore has originated in the mode of selection adopted by the nominating committee. It has been customary for gentlemen to hand in their names and the proposed subjects, on slips of paper, and the committee, without further investigation, have so published in the annual reports. Thus it has happened that appointments have been most injudiciously made, and gentlemen to whom a special duty has been assigned, have been found to know less of that than any other subject. We therefore hoped that no committee of last year would be reappointed or continued, from which no report had been had, and no communication received.

On motion, the nominating committee was unanimously instructed to act upon the suggestions of the chairman, who also stated, that there should be some definite expression of disapprobation as to the course of these gentlemen who had volunteered essays, and had their names reported in the newspapers, and spread over the land, and then paid no attention to the matter.

Dr. Flint, from the committee on prize essays, begged leave to report that they received four dissertations in time for a careful and thorough examination, and two others, quite voluminous, only two days before the meeting of the association. The latter we have felt constrained to exclude altogether from the competition of the present year, on account of the absolute impossibility of reading them with a critical purpose and effect. The others have been carefully examined by all the surviving members of the committee—one estimable associate, Dr. Evans, having been called from all his earthly labors before the active duties of the committee began.

More than one of the four essays we examined exhibited much labor and a commendable scholarship in their preparation—are voluminous, and in some respects very meritorious papers; but in the unanimous judgment of the committee, neither of them possesses the degree and species of merit which should entitle its author to the association prize.

The committee beg leave furthermore to report, that in their opinion, and as the suggestion of their own recent experience, the association should determine in more precise and formal manner than has yet been done, the terms and conditions of competition and of success in the contest for prizes, for the government alike of contestants and the committee of adjudication, and that a committee be now appointed to consider and report upon that subject.

Dr. Gordon, chairman of committee on etiology and pathology of cholera, made a partial report, and asked continuance of time.

On motion, the report was accepted, and referred to committee on publication, and petition for continuance referred to nominating committee.

Dr. J. B. Lindsly, chairman of the committee appointed to inquire into the propriety of dividing the association into sections, for the better performance of its work in considering the various branches of medicine and surgery, recommended the adoption of such a plan, as being indispensably necessary to making this body a working scientific association. They do not deem it necessary to enter into any argument in favor of this plan, it being the one already universally adopted by similar bodies. They would simply recommend for the present, a division into the following sections, as being most suitable to facilitate the transaction of business, viz:

1. Anatomy and Physiology.
2. Chemistry and Materia Medica.
3. Practical Medicine and Obstetrics.
4. Surgery.

The committee do not propose that this subdivision of labor shall in any manner interfere with the regular business of the association as now conducted; but only, that after having assembled each day in general session, each section shall meet separately for the purpose of hearing and discussing papers on such subjects as properly belong to them, and they therefore recommend that the committee of arrangements for the ensuing year, be requested to provide suitable accommodations for the services of these sections, and that each of said sections shall be authorized to make such arrangements as may be required for the proper transaction of its business.

This report was considered and adopted after a very able speech in its support, by Dr. Davis.

Dr. J. B. Flint offered the following resolution:

Whereas, Our brethren of Great Britain are engaged in erecting a monument to the memory of John Hunter, whose invaluable services in behalf of Physiology and Surgery are recognized and honored, as well on this side of the Atlantic as in Europe; and whereas, this association, as the representatives of American medicine, would rejoice in some suitable manner to participate in so grateful a testimonial of gratitude and respect—therefore,

*Resolved*, That a committee of three be appointed, to consider in what manner this participation can best be effected, so as to be acceptable to our British brethren, and consistent with our own means and opportunities of action, with instructions to report at the next annual meeting.

The resolution was adopted, and Drs. Flint, Bowditch, and Shattuck appointed as the committee.

Dr. Harvey Lindsly offered the following:

WHEREAS, Parliamentary rules of order are numerous, complicated, sometimes obscure, and often inapplicable to such a body as the American Medical Association, and whereas, from the nature of the pursuits of medical men, they cannot be familiar with these rules—therefore,

*Resolved*, That a select committee of three members be appointed to prepare a system of rules for the government of this association, as few in number, as concise and as perspicuous as possible, to be reported at the next annual meeting.

This resolution was adopted, and Drs. H. Lindsly, C. G. Comegys, and T. W. Blatchford appointed as a committee.

The nominating committee made the following report:

The next annual meeting to take place at New Haven, on the first Tuesday of June, 1860. Dr. Eli Ives to be junior secretary.

Committee of arrangements—Drs. Charles Hooker, Stephen G. Hubbard, and Benjamin Sullivan, jr., with power to add to their number.

Committee on prize essays—Drs. Worthington Hooker, Conn.; G. C. Shattuck, Mass.; Usher Parsons, R. I.; P. A. Jewett, Conn.; and John Knight, Conn.

The association took up the special order, being the report on medical ethics, to which had been referred the action of the Dubuque Medical Society, which, after debate, was laid over until 12 o'clock to-morrow.

On motion of Dr. H. F. Campbell, a section of meteorology, medical topography, and epidemic diseases, and of medical jurisprudence and hygiene, was added to those already adopted by this association.

The association then proceeded to consider and act upon amendments to the constitution proposed at the last annual meeting, and laid over under the rules.

The following amendment was adopted:

*Resolved*, That the constitution of this association be so amended as to provide that no individual who shall be under sentence of expulsion or suspension from any State or local medical society, of which he may have been a member, shall be received as a delegate to this body, or be allowed any of the privileges of a member, until he shall have been relieved from the said sentence by such State or local society.

The next amendment, lying over from last year, was the proposition of Dr. Kyle of Ohio,

That the constitution of the association be so amended as to prohibit the admission as a delegate or the recognition as a member, of any person who is not a graduate of some respectable medical college.

This amendment was rejected, but, on the question of reconsideration, a long and animated debate ensued. Without arriving at a vote, the association adjourned for dinner.

### AFTERNOON SESSION.

The association was called to order at 3 P. M., Dr. M. F. Askew in the chair. The discussion on the amendment under consideration at the hour of adjournment was renewed.

Dr. Kincaid moved a further amendment, to insert the word "hereafter" after "prohibiting."

The chair ruled the amendment out of order at the present stage, or until the association decides upon the question of reconsideration.

After a long discussion, Dr. Davis of Ind., moved to lay the motion to reconsider on the table, which was carried, 97 yeas, nays not counted—so the amendment stands registered.

The next proposed amendment to the Constitution was that suggested by the New Jersey Medical Society, asking for such changes as would establish a Board of Censors in every judicial district of the Supreme Court, who should examine and grant diplomas to all proper members of the Association.

This was temporarily laid on the table for Dr. Crosby to offer a report of the Medical Teachers' Convention which met on Monday last. He strongly recommended a committee from this body to confer with the Teachers' Committee, and felt great confidence that something beneficial to medical education would be the effect of such conference.

Dr. Comegys moved the appointment of a committee of five to confer with the committee of medical teachers and report at the next annual meeting, provided that no medical teacher be selected on the part of this association.

Dr. T. M. Blatchford, of New York, offered as a substitute the following preamble and resolution:

WHEREAS, of all the subjects which can engage our attention in our associate capacity, that of medical education is paramount; and whereas, harmony of action is essential to success in establishing definite qualifications entitling to admission in our ranks; and whereas, nothing can be gained by hasty action in a matter so vital to our very existence, as a permanent medical institution—therefore,

*Resolved*, That further action be suspended for the present upon the subject of the resolutions offered at the last meeting of the Association, by the chairman of the special committee on medical education, and that a committee consisting of S. W. Butler, of Pennsylvania, L. A. Smith, of New Jersey, Dixi Crosby, of New Hampshire, C. A. Pope, of Mo., and T. Buckler, of Maryland, shall be appointed to confer with the committee appointed at the meeting of medical teachers, to report some plan of action at the next meeting of the association.

This amendment was lost, and the original resolution adopted.

The resolutions from the New Jersey Medical Society were then taken from the table and referred to the committee of conference.

Dr. Davis offered a resolution instructing the same committee to confer with the State Medical Societies, for the purpose of procuring more decisive and uniform action throughout the profession, in carrying into effect the standard of preliminary education adopted by this association at its organization in 1847. This was carried.

Dr. Gibbes, from the committee to examine into a plan of uniform registration of births, marriages and deaths, offered the following report:

They have given the same a careful consideration, and they unanimously recommend that the report be adopted and referred to the committee on publication.

They also recommend that the same committee be continued, with instructions to add to the report in time for publication in the ensuing volume of transactions, a form of registration law which may be likely to answer the requirements of the several States.

Dr. Sayre, of New York, offered the following:

WHEREAS, The medical profession at large have an interest in the character and qualifications of those who are to be admitted as their associates in the profession—therefore,

*Resolved*, That each State Medical Society be requested to appoint annually two delegates for each college in that State, whose duty it shall be to attend the examinations of all candidates for graduation; and that the colleges be requested to permit such delegates to participate in the examination, and vote on the qualifications of all such candidates.

This was, on motion, referred to the committee of conference.

The paper of Dr. Jones, presented at the morning session, was taken from the committee on publication and referred to the committee on prize essays.

Dr. Eve moved to record the name of Dr. Benj. W. Dudley, as a permanent member, which was adopted by a unanimous vote, the delegates all rising to their feet, in token of respect.

Adjourned till to-morrow morning, at 9 o'clock.

THIRD DAY.

THURSDAY, May 9, 1859.

The President called the Association to order at 9 o'clock, and the reading of the minutes of yesterday was dispensed with.

The first business in order was an amendment to the Constitution, laid over from last year, and proposed by Dr. T. L. Mason, of New York, to insert in the first line of the second paragraph of Article 2, after the words "shall receive the appointment from," the words "any medical society permanently organized in accordance with the laws regulating the practice of physic and surgery in the State in which they are situated, and consisting of physicians and surgeons regularly authorized to practice their profession."

Also to add to the sixth paragraph of the same article the words, "but each permanent member of the first class designated in this plan of organization shall be entitled to a seat in the Association on his presenting to this body a certificate of his good standing, signed by the Secretary of the Society to which he may belong at the time of each annual meeting of this body."

Dr. Lyndon A. Smith, of New Jersey, said amendments to the Constitution should be adopted with care, and though, perhaps, that now proposed might be desirable, still, as Dr. Mason, who had proposed it, was not present to explain his views, he moved that the subject be laid over until next year. This suggestion was adopted.

Another constitutional amendment, proposed by Dr. Henry Hartshorne, of Pennsylvania, and laid over from last year under the rules, provides to add to the second article the words, "No one expelled from this Association shall at any time thereafter be received as a delegate or member, unless by a three-fourths vote of the members present at the meeting to which he is sent, or at which he is proposed."

This amendment was adopted.

Another amendment, proposed by J. Berrien Lindsly, of Tennessee, was called up, to omit in article 2 the words, "medical colleges, hospitals, lunatic asylums, and other permanently organized medical institutions in good standing in the United States," and also to omit the words, "The faculty of every regularly constituted medical college or chartered school of medicine shall have the privilege of sending two delegates. The professional staff of every chartered or municipal hospital containing a hundred inmates or more shall have the privilege of sending two delegates, and every other permanently organized medical institution of good standing shall have the privilege of sending one delegate."

This was laid on the table until the next annual meeting.

Dr. Sayre offered the following, which were adopted by acclamation:

*Resolved*, That the thanks of the American Medical Association are eminently due and are hereby presented to the citizens of Louisville, Ky., for the princely hospitality publicly and privately extended to the members of this body during its present session.

*Resolved*, That to the Committee of Arrangements and the Profession of Louisville generally, our tnanks are due for their kind and assiduous attention to the Association, and for the hearty welcome with which they have greeted our Convention in their flourishing city.

After the transaction of some other unimportant routine business,

On motion of Dr. Davis, the Association adjourned to meet at New Haven on the first Tuesday in June, 1860.

The registration book during the day announced the names of Drs. D. G. Thomas, of New York, William S. Cain, of Kentucky, and Peter Allen, R. K. McMeans, and W. R. Kable, of Ohio,—making 305 members in attendance during the session of the Association.—*Semi-Monthly Medical News.*

